# Evaluation under loading detects medial meniscus extrusion in patients with reconstructed anterior cruciate ligament and restricted knee extension

**DOI:** 10.1007/s10396-024-01492-2

**Published:** 2024-09-02

**Authors:** Yosuke Ishii, Atsuo Nakamae, Nekomoto Akinori, Takato Hashizume, Riko Okinaka, Miharu Sugimoto, Kohei Matsumura, Masakazu Ishikawa, Makoto Takahashi, Nobuo Adachi

**Affiliations:** 1 Department of Biomechanics, Graduate School of Biomedical and Health Sciences, Hiroshima University, 1-2-3 Kasumi, Minami-Ku, Hiroshima, 734-8551 Japan; 2 Department of Orthopaedic Surgery, Graduate School of Biomedical and Health Sciences, Hiroshima University, Hiroshima, Japan; 3 Department of Orthopaedic Surgery, Faculty of Medicine, Kagawa University, Kagawa, Japan

**Keywords:** Anterior cruciate ligament reconstruction, Dynamic ultrasound, Knee extension, Meniscus extrusion

## Abstract

**Purpose:**

Some patients who undergo anterior cruciate ligament (ACL) reconstruction experience abnormal mechanical stress in the meniscus. Medial meniscal extrusion (MME) is reflected in the pathological condition of the meniscus, which expands owing to repetitive mechanical stress. Thus, the effect of the reconstructed ACL on increasing MME under weight-bearing conditions remains unclear. This study investigated the effect of ACL reconstruction on meniscal extrusion under non-weight-bearing and weight-bearing conditions.

**Methods:**

Seventeen patients who underwent unilateral ACL reconstruction (ACL group) and 20 age-matched healthy volunteers (control group) were enrolled. Ultrasonography was performed in the supine, standing, and walking positions in preoperative and postoperative ACL patients. MME during walking was evaluated based on the dynamic behavior of extrusion, and kinetic and kinematic data were synchronously obtained. Moreover, the ACL group underwent magnetic resonance imaging (MRI) evaluation at two points: preoperatively and 12 months postoperatively, and the ultrasound findings were compared.

**Results:**

MME in the supine position measured using both ultrasonography and MRI was not significantly different preoperatively and postoperatively in the ACL group. However, postoperative MME and dynamic behavior of extrusion under standing and walking conditions were significantly higher than those in the preoperative state (dynamic behavior: 0.9 ± 0.4 mm preoperatively, 1.2 ± 0.4 mm postoperatively). Moreover, the deficits in knee extension during walking persisted postoperatively and were significantly higher than those in the control group.

**Conclusion:**

MME in patients with ACL reconstruction including meniscus repair was different under mechanical stress compared to the non-weight bearing condition.

## Introduction

Trauma is known to cause knee osteoarthritis (OA) progression due to abnormal mechanical stress on the joint caused by structural destruction. Reconstruction of the anterior cruciate ligament (ACL) is the gold standard treatment for ACL injuries aimed at recovering knee structural stability and function and inhibiting the progression of knee OA [1]. However, some patients experience knee OA progression postoperatively [2–4]. Therefore, it is necessary to develop an evaluation method that can sensitively detect postoperative mechanical stress, thereby leading to appropriate prevention.

The meniscus plays critical roles in shock absolution, joint stability, and load distribution [5], and supports activity of daily living. In particular, the meniscus works at the appropriate location on the tibial plateau, whereas it is often displaced beyond the tibia margin as meniscal dysfunction [6, 7]. Meniscal extrusion reflects abnormal mechanical stress on the femorotibial joint [8] and is a critical indicator of knee OA progression [9, 10]. Meniscal extrusion is known to expand gradually under repetitive mechanical stress [11–13]. However, there is insufficient consensus regarding whether ACL reconstruction affects meniscal extrusion [14, 15]. One explanation for this concern may be different evaluation methods for meniscal extrusion.

Most meniscal extrusions are evaluated using magnetic resonance imaging (MRI) in the supine position, which is the gold standard [14–16]. However, meniscal extrusion responds to mechanical stress and is often observed in weight-bearing conditions, such as standing and walking [17, 18]. This raises concerns that MRI may underestimate meniscal extrusion. Ultrasonography can be used to evaluate different conditions and motions and has been validated [19]. Specific ultrasound techniques are available to detect the dynamics of meniscal extrusion during walking [18, 20]. Thus, comparing postoperative responses between MRI and ultrasonography could provide insight into the biomechanical effects and contribute to the development of an appropriate evaluation method for postoperative patients.

Moreover, the ACL often exhibits different responses in the different knee joint compartments. According to several previous studies, lateral meniscal injuries often occur in conjunction with ACL injuries, whereas medial meniscal injuries are often secondary to knee instability following ACL injury [21, 22]. Therefore, a more sensitive response to meniscal extrusion is expected on the medial side than the lateral side in postoperative patients.

This study aimed to investigate the effects of ACL reconstruction on meniscal extrusion in each compartment using MRI and ultrasonography. We hypothesized that medial meniscal extrusion (MME) in patients who have undergone ACL would differ under mechanical stress during ultrasound evaluation.

## Material and methods

### Participants

This investigation enrolled 17 individuals who had undergone unilateral ACL reconstruction (ACL group, 10 females with a mean age of 31.4 ± 12.0 years) and 20 healthy volunteers (control group, 8 females with a mean age of 26.5 ± 11.0 years). Patients in the ACL group underwent two evaluations: preoperatively (baseline) and approximately 12 months postoperatively. The participant selection criteria were based on their ability to walk smoothly, whereas the exclusion criteria included individuals with (1) bilateral ACL injury, (2) neurological disorder, (3) history of contralateral orthopedic surgery, and (4) use of the bone-patella-tendon-bone reconstruction technique. Table 1 presents the demographic data.

**Table 1:** Demographic data of participants

	ACL reconstruction	Control	p-value
Knees	17	20	
Sex (M:F)	7:10	12:8	
Age (years)	31.4 ± 12.0	26.5 ± 11.0	0.22
BMI (kg/m^2^)	23.6 ± 2.4	22.3 ± 1.9	0.08

ACL, anterior cruciate ligament

Sex (M:F), male:female

BMI, body mass index

Values are expressed as means ± standard deviation

This study was approved by the Ethics Department of our institution and adhered to the Declaration of Helsinki (E2021-2498–02). Informed consent was obtained from all participants.

### ACL reconstruction and evaluation of meniscal quality

Two skilled surgeons (N.A. and A.N.) performed ACL reconstruction and augmentation. Patients underwent augmentation using a semitendinosus tendon graft if approximately one-third or more of the original ACL remained intact, thereby establishing a ligamentous bridge between the tibia and the femur. In patients with insufficient ACL remnants, reconstruction was performed using a semitendinosus tendon graft. The decision between the single- or double-bundle technique was based on the graft diameter and sizes of the tibia and femur. Moreover, an arthroscopic evaluation was simultaneously performed during ACL reconstruction, and the meniscal injury type and location in the knee were recorded. If there were complications of meniscal injury, including instability, the meniscus was subjected to suture fixation using the all-inside or inside-out technique.

Postoperatively, a soft knee brace was applied to immobilize the reconstructed knee for 3 days in cases of standalone ACL reconstruction and for 2 weeks in cases involving meniscal repair. After discharge, the patient underwent regular monitoring at the outpatient clinic and continued to use a brace until the third postoperative month. Running activities were authorized at the 4.5-month mark postoperatively, and the gradual introduction of non-contact sports and training was sanctioned at the 6-month postoperative stage.

### Evaluation of knee alignment and range of motion

Baseline radiographic assessment was performed on ACL participants while bearing weight on the whole leg. Knee varus or valgus alignment was examined using the hip-knee-ankle angle (HKAA), with positive values indicating valgus alignment of the knee joint. To minimize potential bias, a single orthopedic surgeon (A.N.), blinded to the clinical data, performed the radiographic evaluation. Moreover, the knee range of motion in the sagittal plane was recorded at baseline and postoperatively in the supine position.

### Evaluation *of meniscus* extrusion on MRI

For patients in the ACL group, MRI (Ingenia 3.0 T, Philips) examinations were performed with the patient in the supine position at both baseline and 12 months postoperatively to assess the reconstructive ligament and meniscus extrusion. Using methods described in a previous study [14], meniscal extrusion was evaluated to identify the distance from the tibial line (excluding osteophytes) to the peripheral aspect of the meniscus on midcoronal slices on proton density-weighted images.

### Gait analyses

The walking kinematics and kinetics of the participants were assessed using a combination of 16 cameras (VICON612; Vicon Motion Systems, Oxford, UK) and eight force plates (AMTI, Watertown, MA, USA) at sampling rates of 100 and 1000 Hz. Sixteen reflective markers aligned with the plug-in-gait lower-body marker set were placed on anatomical landmarks. The participants were instructed to walk at a comfortable speed, and this process was repeated twice. The single-stance phase was identified from the heel contact to toe-off, and these events were determined based on a ground reaction force threshold of 10 N.

The knee angle and moment during the single-stance phase were calculated using Nexus 1.8.5, and the output data were normalized to 100 data points. The primary focus was on sagittal knee motion, including knee flexion and extension angles and peak knee flexion and extension moments. The flexion range was calculated as the difference in the knee flexion angle between the initial contact and the maximum point in the early stance phase. These processes were performed using MATLAB R 2020a (MathWorks).

### Ultrasound evaluation *of meniscus* extrusion

**Fig. 1:**
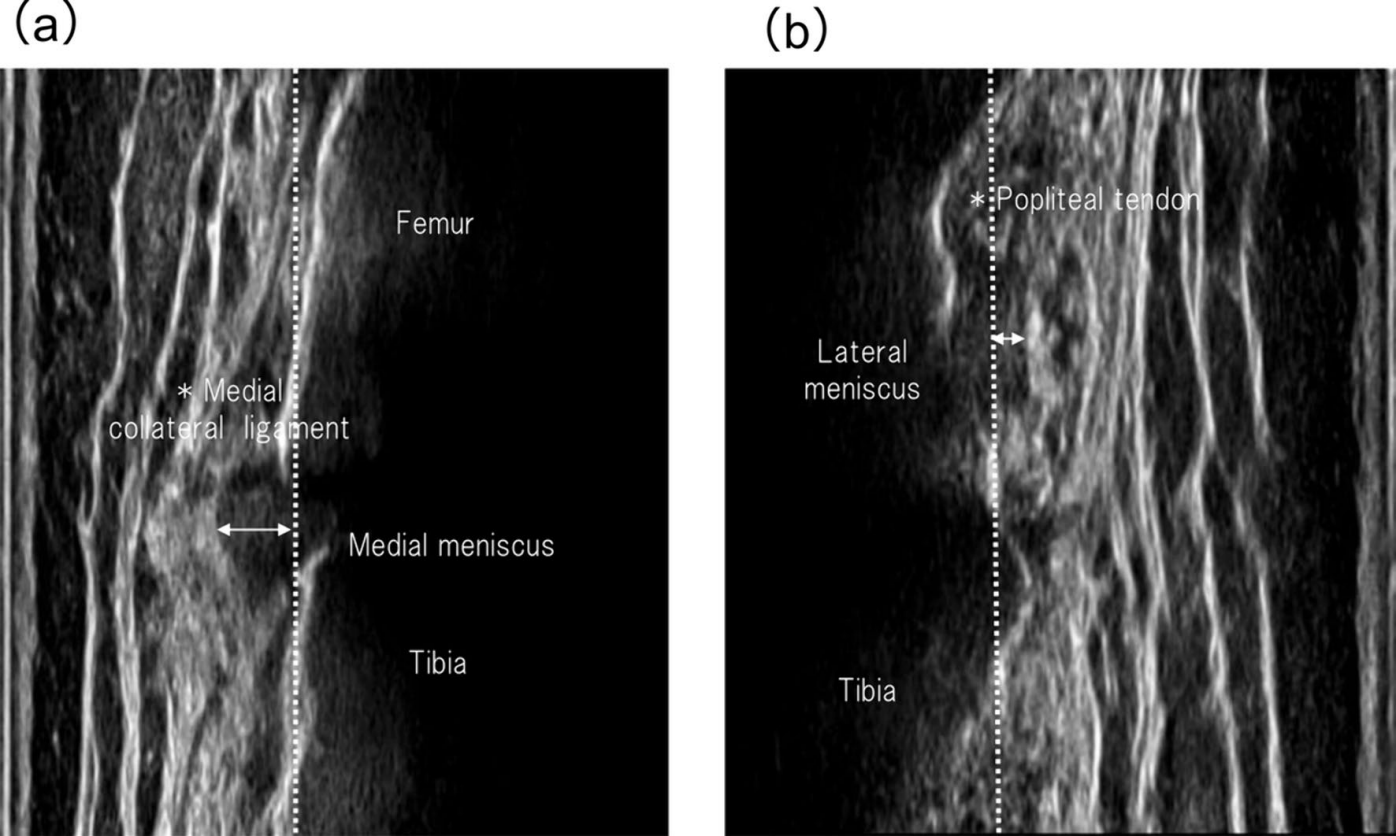
Representative ultrasound images. Coronal images depict the medial **a** and lateral **b** menisci, with * indicating the medial collateral ligament and popliteal tendon as landmarks. White dashed lines represent the reference line based on the tibial plate and arrows indicate the extent of meniscal extrusion

The meniscus was evaluated using ultrasonography with a unique prototype linear array transducer at 3–11 MHz (SNiBLE; KONICA MINOLTA, Japan). The medial and lateral menisci were observed during full extension of the knee. The transducer was positioned longitudinally in the medial joint space to capture triangular morphological images of the medial meniscus. Image acquisition targeted the point where the medial collateral ligament was most distinct, confirming the boundary between the medial meniscus edge and deep medial collateral ligament **(**Fig. 1a**)**. The transducer was placed longitudinally on the lateral meniscus in the lateral knee joint space with the participant’s knee fully extended. Visualization of the origin of the popliteal tendon and flat cortex on the tibial plate has been confirmed in previous studies [23, 24] **(**Fig. 1b**)**.

Meniscal evaluation was performed by using static images in the supine and standing positions. The measurement of MME or lateral meniscal extrusion (LME) involved determining the distance from the cortical line of the tibial plate to the outermost edge of the meniscus, a definition established in a previous study [24, 25] **(**Fig. 1**)**, which was calculated using Kinovea software.

**Fig. 2:**
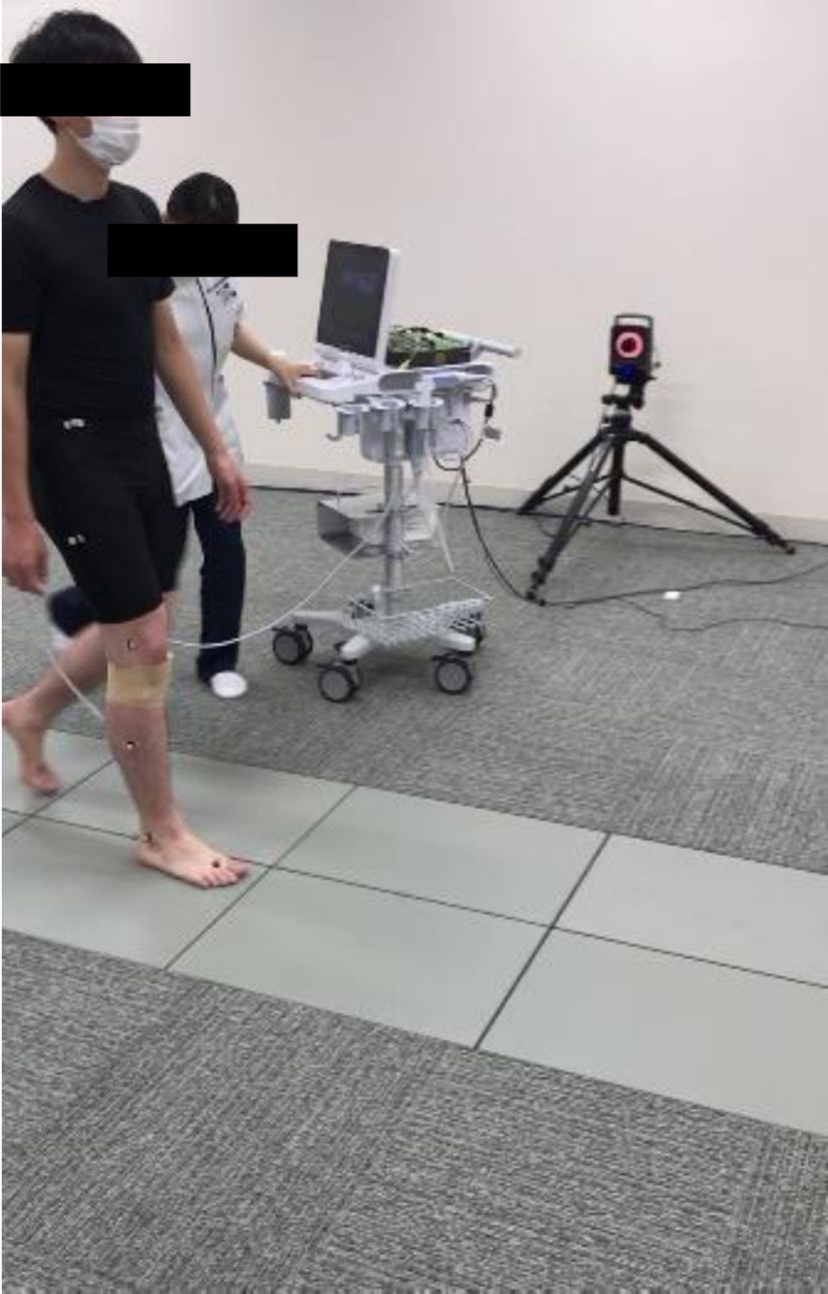
Dynamic ultrasonographic imaging. The measurement image of dynamic evaluation of meniscus extrusion during walking

**Fig. 3:**
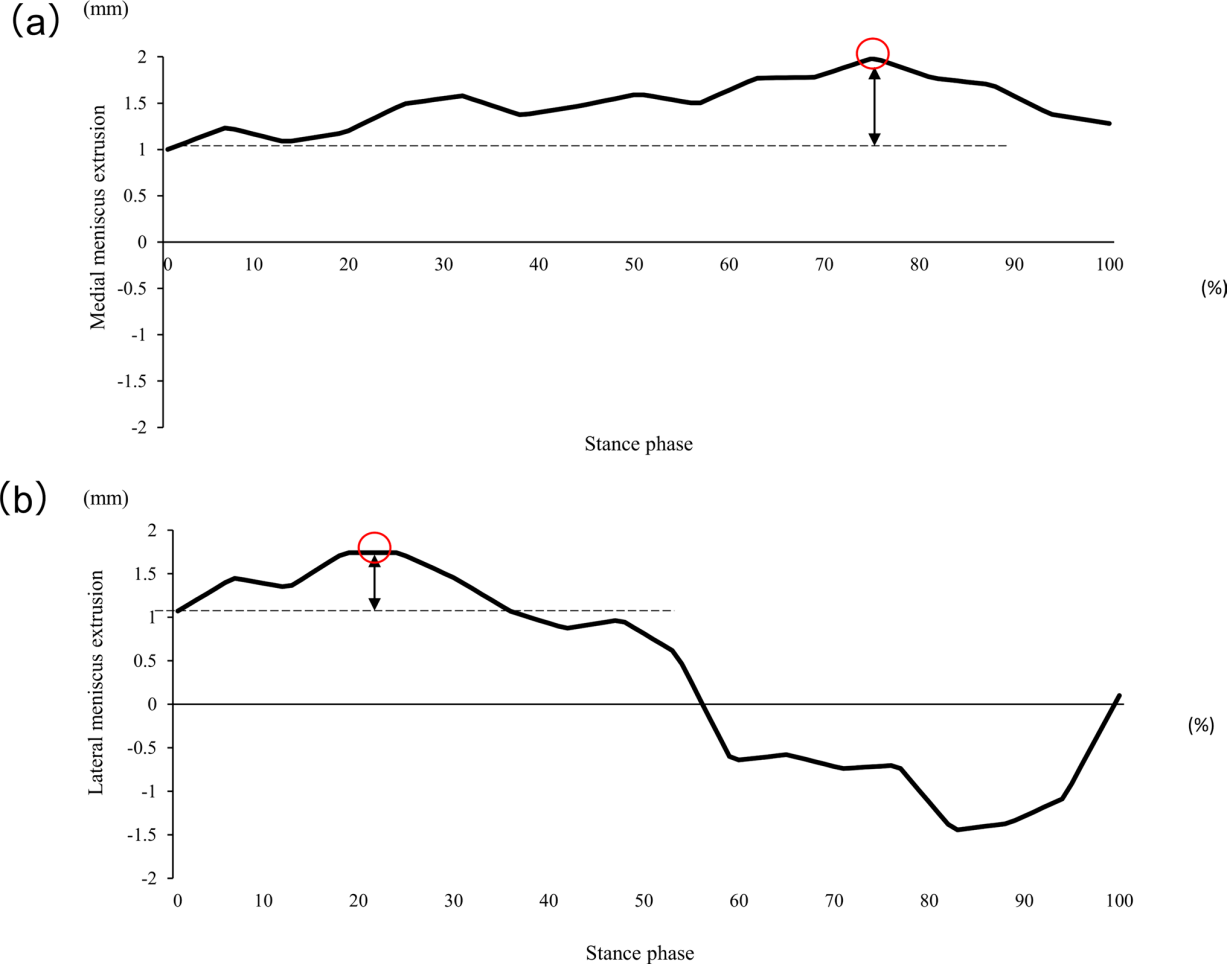
Waveform of meniscal extrusion during walking. Waveform of meniscal extrusion observed during walking. Representative images depicting the medial **a** and lateral **b** sides. These waveforms were constructed on the basis of approximately 25 consecutive meniscal extrusion values in sequential images. The red circle and dotted line indicate the positions representing maximum meniscal extrusion and the initial point, respectively. The double arrow shows the dynamic behavior of meniscal extrusion

Walking was conducted on a 5-m straight road **(**Fig. 2**)**. The dynamics of the meniscus were recorded in video mode on ultrasound, which was synchronized with motion analysis using an electrocardiogram. Participants were instructed to walk at a comfortable speed. The analysis focused on the single-stance phase. Approximately 25 ultrasound images were captured during this procedure, and meniscal extrusion was calculated for each image, excluding the last three. Waveforms of meniscal extrusion during walking were obtained using continuous values. To compare the waveforms across participants with varying numbers of images, the stance phase was standardized to 100 data points. These established methodologies are highly reliable [24, 25]. Furthermore, meniscal extrusion behavior was indicated by the distance between the initial (zero) and maximum values **(**Fig. 3**)**.

### Statistical analysis

The entire dataset was assessed for normality. Demographic information, meniscal values of both MME and LME, and gait parameters were compared between the groups and subgroups, including those with and without meniscal tears, using either the non-paired *t*-test or Mann–Whitney *U* test. Comparisons of the MME, LME, and gait parameters between the baseline and postoperative periods were conducted using a paired t-test or Wilcoxon rank-sum test. Statistical analyses were performed using SPSS version 23 (IBM, Armonk, NY, US), and statistical significance was set at *p* < 0.05.

To assess the impact of ACL reconstruction without meniscus repair on MME behavior during walking in the ultrasound evaluation, a power analysis was performed using G-power. The effect size was represented as the difference in preoperative and postoperative MME behavior during walking in 10 patients with only ACL reconstruction. The results indicated an effect size of 1.42 and a power of 97.8%.

## Results

### Demographic data for participants and meniscus quality

The participants’ characteristics are presented in Table 1. The mean waiting period was 114.8 ± 55.8 days. For patients in the ACL group, the HKAA was 0.6 ± 1.9° at baseline. A few restricted ranges of motion were observed at baseline and postoperatively (extension: -2.6 ± 5.2° preoperatively, -1.7 ± 2.8° postoperatively; flexion: 134.7 ± 4.8° preoperatively, 138.2 ± 3.4° postoperatively).

Regarding meniscal quality, seven patients experienced complications of medial meniscal injury. Six complications were longitudinal tears in the posterior section and one was in the middle section of the medial meniscus. Seven patients experienced complications due to lateral meniscal injury. Four patients had longitudinal tears in the posterior section, and three patients had lateral tears in the middle or posterior horn of the lateral meniscus. Therefore, there was no patient with meniscus posterior root tear.

### Meniscus extrusion in the supine position on MRI and ultrasonography

On MRI, MME did not significantly differ between examinations (1.7 ± 1.2 mm preoperatively, 1.8 ± 1.5 mm postoperatively). Moreover, LME also showed no significant difference (-1.7 ± 2.3 mm preoperatively, -1.8 ± 2.1 mm postoperatively).

**Fig. 4:**
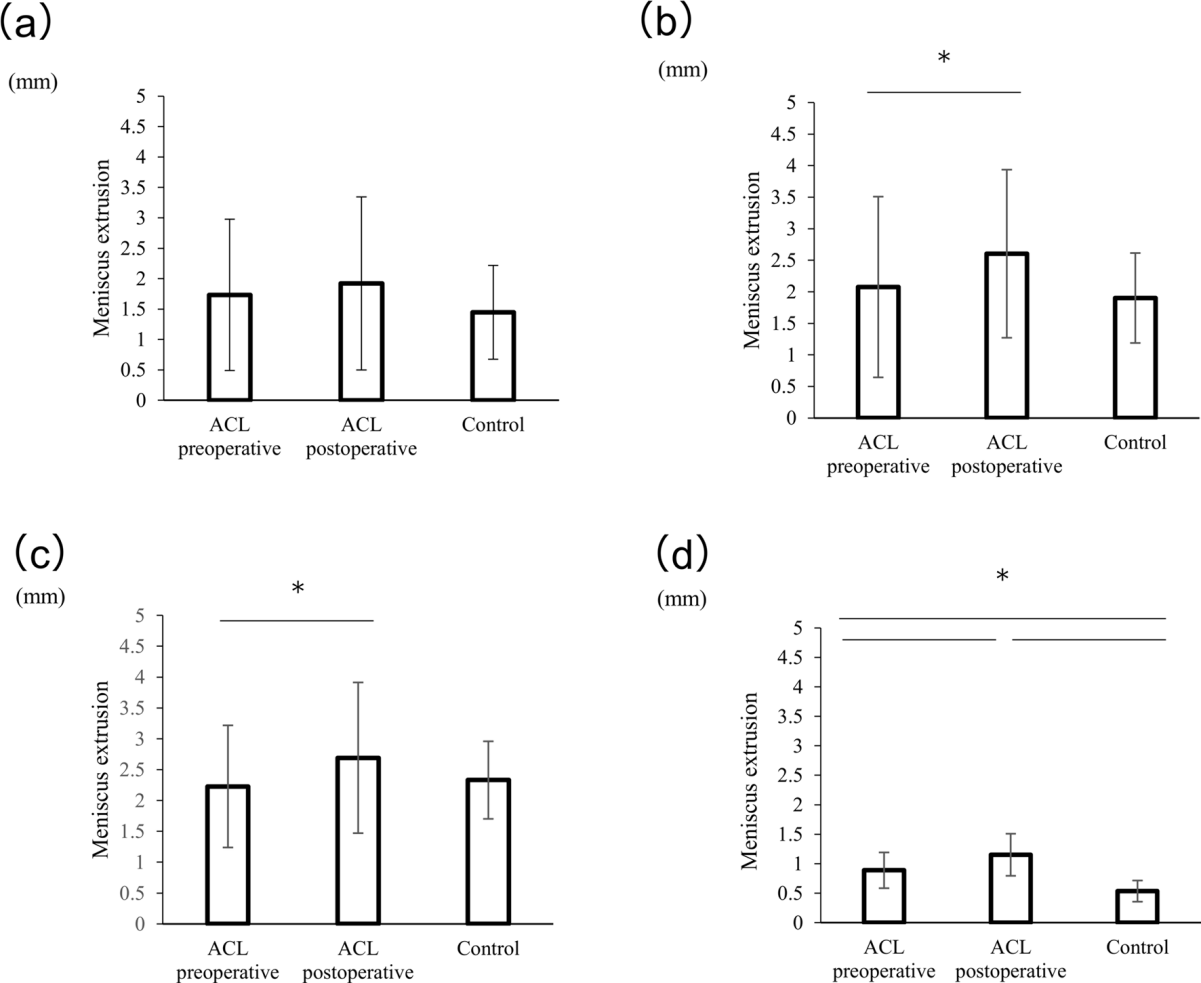
The extrusion of medial meniscus parameters on ultrasound. Preoperative and postoperative values are shown. MME on ultrasound images in the supine **a**, standing **b**, and walking **c** positions. The meniscal extrusion behavior during walking is shown (**d**). These values are presented as mean ± standard deviation, with * denoting a significant difference between groups or at follow-up (p < 0.05)

**Fig. 5:**
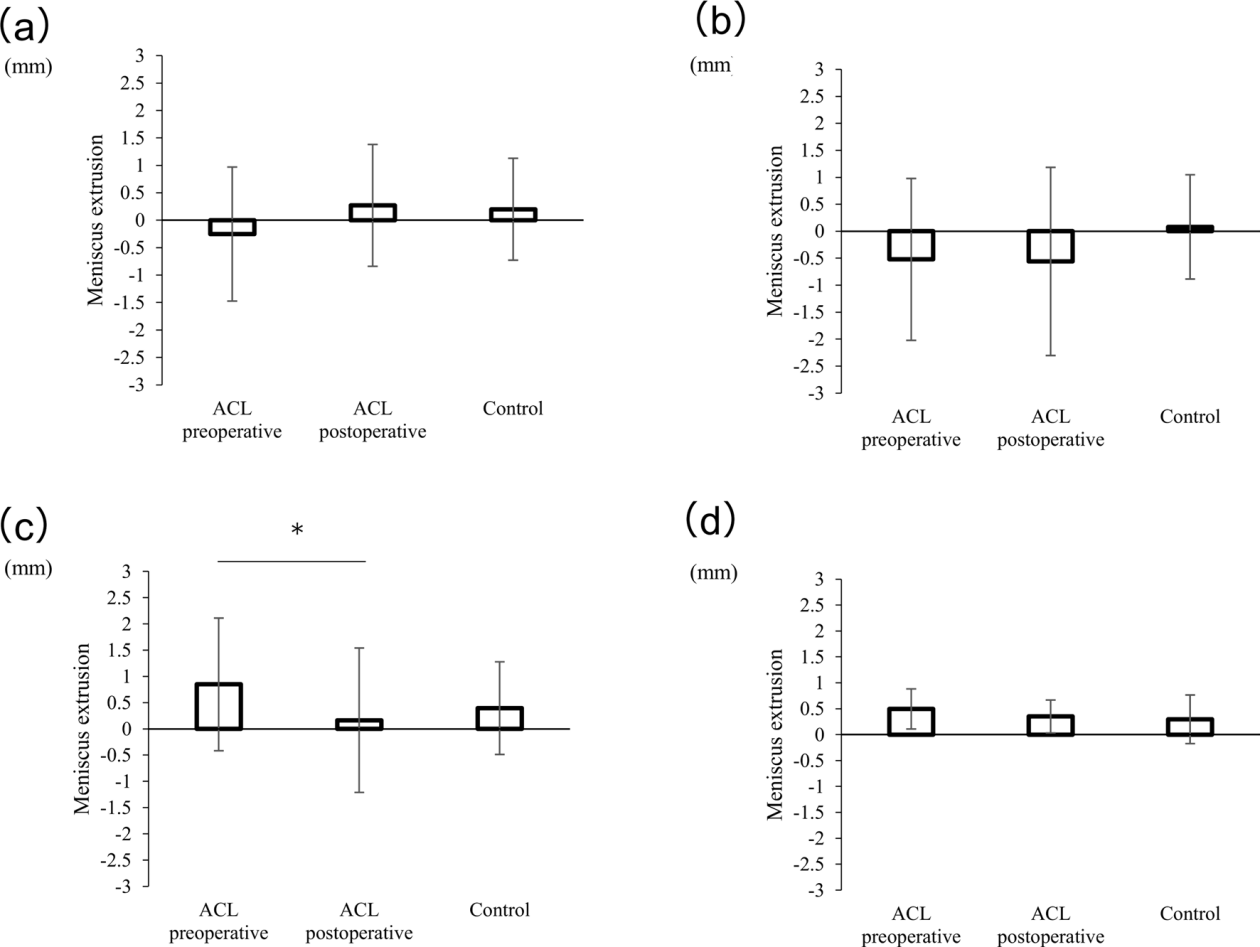
The extrusion of lateral meniscus parameters on the ultrasound. Preoperative and postoperative values are shown. LME on ultrasound images in the supine (**a**), standing (**b**), and walking (**c**) positions. The meniscal extrusion behavior during walking is shown (**d**). These values are presented as mean ± standard deviation, with * denoting a significant difference between follow-up visits (p < 0.05)

On ultrasonography, MMEs and LMEs did not show significant differences between the groups or pre- and postoperatively on each side (MME: 1.9 ± 1.4 mm preoperatively, 2.1 ± 1.1 mm postoperatively, 1.5 ± 0.8 mm for control; LME: -0.3 ± 1.3 mm preoperatively, 0.3 ± 1.1 mm postoperatively, 0.2 ± 0.9 mm for control) **(**Figs. 4, 5a**)**.

### Standing and walking meniscus extrusion using ultrasonography

Under the standing condition, postoperative MME was significantly higher than that at baseline, but not that in the control group (MME: 2.1 ± 1.4 mm preoperatively, 2.6 ± 1.3 mm postoperatively, 1.9 ± 0.7 mm for control; preoperative vs postoperative: *p* = 0.015) **(**Fig. 4b**)**. LME was not significantly different between groups or at follow-up (– 0.5 ± 1.5 mm preoperatively, – 0.6 ± 1.7 mm postoperatively, 0.1 ± 0.9 mm for control) **(**Fig. 5b**).**

Under the walking condition, postoperative maximum MME was significantly higher than that at baseline, but not that in the control group (2.2 ± 1.0 mm preoperatively, 2.7 ± 1.2 mm postoperatively, 2.3 ± 0.6 mm for control; preoperative vs postoperative: *p* = 0.015). The postoperative behavior of the MME was significantly better than that at baseline and in the control group. Moreover, the behavior of the MME at baseline in the ACL group was significantly higher than that of the control group. (0.9 ± 0.3 mm preoperatively, 1.2 ± 0.4 mm postoperatively, 0.5 ± 0.2 mm for control; preoperative vs postoperative: p = 0.019; preoperative vs control: p = 0.001; postoperative vs. control: p = 0.001) **(**Fig. 4c, d**)**. In contrast, the maximum postoperative LME was significantly lower than that at baseline, but not that in the control group (0.8 ± 1.3 mm preoperatively, 0.2 ± 1.4 mm postoperatively, 0.3 ± 0.9 mm for control; preoperative vs postoperative: p = 0.03). The behavior of LME was not significantly different between groups or at postoperative follow-up (0.5 ± 0.4 mm preoperatively, 0.4 ± 0.3 postoperatively, 0.3 ± 0.5 mm for control) **(**Fig. 5c, d**).**

### Time-course change in kinematics and kinematics during walking

In the ACL group, the knee angles, moments, and GRF did not show significant differences between ACL examinations (Table 2**)**.

**Table 2:** Between-group comparisons of kinetic and kinematic parameters during walking

	ACL reconstruction			Control	Vs preoperative	Vs postoperative
	Preoperative	Postoperative	p-value ^a^		p-value ^b^	p-value ^c^
Initial knee flexion angle (°)	7.0 ± 6.8	9.5 ± 4.9	0.202	0.3 ± 4.2	0.002	0.001
Maximum knee flexion angle (°)	15.2 ± 6.4	17.9 ± 3.8	0.072	9.5 ± 4.7	0.007	0.001
Knee range of motion (°)	8.1 ± 2.7	8.4 ± 3.8	0.778	8.7 ± 2.6	0.531	0.795
Maximum knee extension angle (°)	– 11.0 ± 7.7	– 9.0 ± 5.4	0.37	– 1.4 ± 3.8	0.001	0.001
Knee flexion moment (Nm/kg)	0.5 ± 0.3	0.5 ± 0.2	0.475	0.5 ± 0.2	0.819	0.689
Knee extension moment (Nm/kg)	– 0.1 ± 0.2	– 0.1 ± 0.2	0.5	0.01 ± 0.2	0.037	0.057
Vertical ground reaction force (N)	672.5 ± 107.8	691.4 ± 107.2	0.217	674.5 ± 101.9	0.954	0.637

ACL, anterior cruciate ligament

*p-value *^*a*^*,* preoperative vs. postoperative patients; *p-value *^*b*^*,* preoperative vs. controls; *p-value *^*c*^*,* postoperative vs. controls

The data were obtained with medial meniscus extrusion, and values are expressed as means ± standard deviation

At both baseline and postoperatively, the knee flexion angles were significantly higher than those in the control group (15.2 ± 6.4°preoperatively, 17.9 ± 3.8°postoperatively, 9.5 ± 4.7 for control; preoperative vs control: p = 0.007; postoperative vs control: p = 0.001). The knee extension angle was significantly lower than those in the control group (knee extension angle: – 11.0 ± 7.7°preoperatively, – 9.0 ± 5.4°postoperatively, – 1.4 ± 3.8° for control; preoperative vs control: p = 0.001; postoperative vs control: p = 0.001). The knee extension moment at baseline was significantly lower than that in the control group (knee extension moment: – 0.1 ± 0.2 Nm/kg preoperatively, – 0.1 ± 0.2 Nm/kg postoperatively, 0.01 ± 0.2 Nm/kg for control; preoperative vs control: p = 0.037; postoperative vs control: p = 0.057). (Table 2**)**.

### Comparison *of meniscus* dynamics between subgroups with meniscus repair

In the subgroup with medial meniscal repair, no significant difference was observed in MME parameters between baseline and postoperative values. On the other hand, in the subgroup without meniscus repair, the behavior of MME only during walking was higher postoperatively than that at baseline (0.8 ± 0.2 mm preoperatively, 1.2 ± 0.3 mm postoperatively, *p* = 0.001). Regarding LME, in the subgroup without meniscus repair, the behavior of LME only during walking was lower postoperatively than that at baseline (0.6 ± 0.3 mm preoperatively, 0.3 ± 0.3 mm postoperatively, *p* = 0.016).

### Time-course change in kinematics and kinematics during walking in meniscus repair subgroup

In the subgroup without meniscus repair, initial and maximum knee flexion angles were greater postoperatively than those at baseline (initial knee flexion: 6.0 ± 7.3° preoperatively, 11.4 ± 4.7° postoperatively, preoperative vs postoperative: p = 0.014; maximum knee flexion: 13.4 ± 6.7° preoperatively, 17.4 ± 4.2° postoperatively, preoperative vs postoperative: p = 0.021), but not those in the meniscus repair subgroup.

Kinematics and kinematic parameters did not differ significantly between the subgroups at baseline. On the other hand, postoperatively in the subgroup without medial meniscus repair, initial knee flexion angle was greater than that in the subgroup with meniscus repair (without meniscus repair: 11.4 ± 4.7°, with meniscus repair: 6.7 ± 3.7°, p = 0.015), and the range of motion occurring in the early stance phase also exhibited a lower value than that in the ACL reconstruction group with meniscus repair (without meniscus repair: 5.9 ± 1.8°, with meniscus repair: 12.0 ± 3.0°, *p* = 0.002) (Table 3**)**.

**Table 3:** Comparison of kinetic and kinematic parameters during walking among subgroups

	ACL reconstruction with repair			ACL reconstruction without repair		
	Preoperative	Postoperative	p-value	Preoperative	Postoperative	p-value
Initial knee flexion angle (°)	8.6 ± 5.7	6.7 ± 3.7	0.57	6.0 ± 7.3	11.4 ± 4.7*	0.014
Maximum knee flexion angle (°)	17.7 ± 4.7	18.7 ± 3.0	0.746	13.4 ± 6.7	17.4 ± 4.2	0.021
Knee range of motion (°)	9.2 ± 3.3	12.0 ± 3.0*	0.093	7.5 ± 1.9	5.9 ± 1.8	0.159
Maximum knee extension angle (°)	– 13.5 ± 4.8	– 9.2 ± 5.8	0.291	– 9.2 ± 8.7	– 8.8 ± 5.0	0.889
Knee flexion moment (Nm/kg)	0.6 ± 0.2	0.5 ± 0.2	0.632	0.4 ± 0.2	0.5 ± 0.2	0.229
Knee extension moment (Nm/kg)	– 0.3 ± 0.2	– 0.2 ± 0.1	0.304	– 0.1 ± 0.2	– 0.1 ± 0.1	0.981
Vertical ground reaction force (N)	664.4 ± 65.6	694.1 ± 97.2	0.239	678.1 ± 129.1	689.6 ± 113.8	0.581

ACLR, anterior cruciate ligament reconstruction. Values are expressed as means ± standard deviation

*p-values* reflect differences between preoperative and postoperative patients

^*^ Significantly higher values than those postoperatively between the repair and no-repair subgroups (*p* < 0.05)

## Discussion

This study found that the medial meniscus and its behavior observed under standing and walking conditions worsened in patients with ACL reconstruction using ultrasonography, but not in the supine position, as observed on MRI. This response was observed only in the medial meniscus. These results were consistent with our hypotheses.

Our data showed that MME under standing and walking conditions showed significant postoperative changes observed on ultrasonography, but not in the supine position observed on both MRI and ultrasonography. Thus, MME was sensitively detected, depending on the mechanical stress. In general, the MME value immediately varies depending on the measurement condition, i.e., it increases under the weight-bearing condition compared to the non-weight-bearing condition [13, 26]. In particular, it is obvious that the meniscus is involved in higher stress during walking [20, 27, 28], and the dynamics of meniscal extrusion during walking worsen MME under weight-bearing conditions over time. In this study, they remain involved in aberrant kinematics, such as deficient knee extension during walking. These factors have a greater impact on the cumulative stress in the knee joint, depending on the tibial malalignment [29], which may explain the increased MME in postoperative patients. Therefore, these results and those of previous studies could explain why evaluation only under the non-weight conditions underestimates abnormal pathological mechanics of the knee joint in patients who have undergone ACL reconstruction.

Although a patient who underwent ACL reconstruction sustained an injury to the meniscus that was almost equal on both the medial and lateral sides, worsening of meniscal extrusion was observed only on the medial side in the standing and walking motions. Thus, postoperative changes in MME may be caused by another factor without morphological changes. The medial meniscus acts as a second stabilizer in the anteroposterior direction of the knee joint [30–32], especially in the sagittal range of motion. Moreover, the aberrant kinematics associated with the stiffened knee strategy may cause mechanical stress on the knee joint [33]. In this study, the patient remained deficient in knee extension during walking, in which it was speculated that the medial meniscus required extreme work, and the patient experienced repeated abnormal stress during walking, even in daily living. In fact, there is a high incidence of medial meniscus lesions after ACL injury in chronic patients [21, 22], which often involves complications such as deficient knee extension. Moreover, cumulative mechanical stress immediately causes MME, but not LME, even in healthy knees [34]. Therefore, these previous studies support our hypothesis that postoperative patients have worsening MME owing to abnormal cumulative mechanical stress.

Interestingly, in the subgroup without meniscus repair, the behavior of MME during walking was higher postoperatively than at baseline, whereas the subgroup with meniscal repair did not show worsening dynamics of MME. However, Katagiri et al. showed that ACL reconstruction with an additional approach for longitudinal tears resulted more frequently in meniscal extrusion measured 3 months postoperatively [14]. Therefore, our results are not in accord with the claim that the dynamics of MME might more strongly affect pathological meniscal conditions. One possible explanation is that other effects may mask the effects of meniscal repair. In this study, participants were followed-up at 12-months postoperatively, where their knee might be exposed to the mechanical stress in daily living for a relatively longer period than in the previous study. Moreover, the subgroup without medial meniscus repair had deficits in knee extension and range of motion during walking compared with patients with medial meniscus repair. This postoperative deficit in knee extension was caused by multiple factors, such as knee muscle strength, soft tissue fibrosis, capsular adhesions, and sex, whereas meniscus complications were not included [35]. Therefore, although this study cannot refer to the cause of deficit knee extension, it provides valuable information on the mechanism by which the dynamics of MME in postoperative ACL reconstruction could also contribute to aberrant kinematics rather than meniscal pathology itself.

In general, a traumatic event of the knee joint is a known risk factor for progression of knee OA due to abnormal mechanical stress [36]. ACL reconstruction is the gold standard approach for patients with ACL injuries to achieve high recovery of function [37, 38] and prevent knee OA progression [2]. Unfortunately, some patients show knee OA progression after ACL reconstruction [2, 4, 39, 39]. Therefore, it is necessary to detect abnormal mechanical stress to develop appropriate approaches. Several studies have argued that accelerated progression of knee OA is associated with worsening of MME underlying the abnormal dynamics of extrusion [40, 41]. Our findings showed worsening of MME and dynamic extrusion, especially during walking. Interestingly, extension restrictions were not observed in the supine position either at baseline or postoperatively, whereas these occurred during walking as abnormal kinematics. Therefore, our findings indicate that evaluation under non-weight-bearing conditions alone underestimates the mechanopathology due to abnormal kinematics in patients who have undergone ACL reconstruction.

This study had certain limitations. First, the study involved a small sample size; therefore, analysis of features such as the type of ACL reconstruction, meniscal condition, and sex disparities among the patients was insufficient. These factors could affect our findings. Second, the pathological meniscus condition or approach might distort findings. Therefore, we performed subgroup analysis to detect the pure effect of the ACL reconstruction on MME and indicate the postoperative aggravation of MME with abnormal kinetics. However, it raises concerns that the small sample size does not sufficiently reflect the general population of ACL patients. Third, this study did not provide detailed information about kinetics, such as tibial movement in the anterior-to-posterior direction, because of the application of limited motion capture techniques for synchronization with ultrasonography. Fourth, this study did not include MRI and radiographic data of healthy volunteers; therefore, it was not possible to define a healthy knee. Future studies are needed to add a detailed evaluation of kinetic motion and meniscus information and to provide a detailed analysis with a sufficient sample size.

## Conclusion

MME in patients who had undergone ACL reconstruction was different under mechanical stress compared to that under non-weight-bearing conditions. Static evaluation under non-weight-bearing conditions may underestimate the MME underlying the abnormal mechanical stress in patients who have undergone ACL reconstruction, including a deficit in knee extension during walking.

## Data Availability

The datasets generated and/or analysed during the current study are available from the corresponding author on reasonable request.

## References

[1] Paschos NK. Anterior cruciate ligament reconstruction and knee osteoarthritis. World J Orthop. 2017;8:212–7. 28361013 10.5312/wjo.v8.i3.212 PMC5359756

[2] Ajuied A, Wong F, Smith C, et al. Anterior cruciate ligament injury and radiologic progression of knee osteoarthritis: a systematic review and meta-analysis. Am J Sports Med. 2014;42:2242–52. 24214929 10.1177/0363546513508376

[3] Øiestad BE, Engebretsen L, Storheim K, et al. Knee osteoarthritis after anterior cruciate ligament injury: a systematic review. Am J Sports Med. 2009;37:1434–43. 19567666 10.1177/0363546509338827

[4] Luc B, Gribble PA, Pietrosimone BG. Osteoarthritis prevalence following anterior cruciate ligament reconstruction: a systematic review and numbers-needed-to-treat analysis. J Athl Train. 2014;49:806–19. 25232663 10.4085/1062-6050-49.3.35 PMC4264654

[5] Walker PS, Arno S, Bell C, et al. Function of the medial meniscus in force transmission and stability. J Biomech. 2015;48:1383–8. 25888013 10.1016/j.jbiomech.2015.02.055

[6] Breitenseher MJ, Trattnig S, Dobrocky I, et al. MR imaging of meniscal subluxation in the knee. Acta Radiol Stockh Swed. 1987;1997(38):876–9. 10.1080/02841859709172428 9332248

[7] Gale DR, Chaisson CE, Totterman SM, et al. Meniscal subluxation: association with osteoarthritis and joint space narrowing. Osteoarthritis Cartilage. 1999;7:526–32. 10558850 10.1053/joca.1999.0256

[8] Marzo JM, Gurske-DePerio J. Effects of medial meniscus posterior horn avulsion and repair on tibiofemoral contact area and peak contact pressure with clinical implications. Am J Sports Med. 2009;37:124–9. 18815238 10.1177/0363546508323254

[9] Berthiaume M-J, Raynauld J-P, Martel-Pelletier J, et al. Meniscal tear and extrusion are strongly associated with progression of symptomatic knee osteoarthritis as assessed by quantitative magnetic resonance imaging. Ann Rheum Dis. 2005;64:556–63. 15374855 10.1136/ard.2004.023796 PMC1755443

[10] Swamy N, Wadhwa V, Bajaj G, et al. Medial meniscal extrusion: Detection, evaluation and clinical implications. Eur J Radiol. 2018;102:115–24. 29685524 10.1016/j.ejrad.2018.03.007

[11] Furumatsu T, Kodama Y, Kamatsuki Y, et al. Meniscal Extrusion Progresses Shortly after the Medial Meniscus Posterior Root Tear. Knee Surg Relat Res. 2017;29:295–301. 29172390 10.5792/ksrr.17.027 PMC5718799

[12] Ishii Y, Hashizume T, Okamoto S, et al. Cumulative knee adduction moment during jogging causes temporary medial meniscus extrusion in healthy volunteers. J Med Ultrason. 2001;2023(50):229–36. 10.1007/s10396-023-01288-w PMC11976817 36800121

[13] Ishii Y, Ishikawa M, Kurumadani H, et al. Increase in medial meniscal extrusion in the weight-bearing position observed on ultrasonography correlates with lateral thrust in early-stage knee osteoarthritis. J Orthop Sci Off J Jpn Orthop Assoc. 2020;25:640–6. 10.1016/j.jos.2019.07.003 31350063

[14] Katagiri H, Miyatake K, Nakagawa Y, et al. The effect of a longitudinal tear of the medial meniscus on medial meniscal extrusion in anterior cruciate ligament injury patients. Knee. 2019;26:1292–8. 31519329 10.1016/j.knee.2019.07.019

[15] Narazaki S, Furumatsu T, Tanaka T, et al. Postoperative change in the length and extrusion of the medial meniscus after anterior cruciate ligament reconstruction. Int Orthop. 2015;39:2481–7. 25693884 10.1007/s00264-015-2704-z

[16] Costa CR, Morrison WB, Carrino JA. Medial meniscus extrusion on knee MRI: is extent associated with severity of degeneration or type of tear? AJR Am J Roentgenol. 2004;183:17–23. 15208101 10.2214/ajr.183.1.1830017

[17] Ko C-H, Chan K-K, Peng H-L. Sonographic imaging of meniscal subluxation in patients with radiographic knee osteoarthritis. J Formos Med Assoc Taiwan Yi Zhi. 2007;106:700–7. 17908659 10.1016/S0929-6646(08)60031-5

[18] Ishii Y, Nakashima Y, Ishikawa M, et al. Dynamic ultrasonography of the medial meniscus during walking in knee osteoarthritis. Knee. 2020;27:1256–62. 32711889 10.1016/j.knee.2020.05.017

[19] Marcello H. Nogueira-Barbosa, Gregio-Junior E, Lorenzato MM, Guermazi A, et al. Ultrasound assessment of medial meniscal extrusion: a validation study using MRI as reference standard. AJR Am J Roentgenol. 2015;204:584–8. 10.2214/AJR.14.12522 25714289

[20] Ishii Y, Ishikawa M, Nakashima Y, et al. Dynamic ultrasound reveals the specific behavior of the medial meniscus extrusion in patients with knee osteoarthritis. BMC Musculoskelet Disord. 2023;24:272. 37038148 10.1186/s12891-023-06361-6 PMC10084641

[21] Cipolla M, Scala A, Gianni E, et al. Different patterns of meniscal tears in acute anterior cruciate ligament (ACL) ruptures and in chronic ACL-deficient knees. Classification, staging and timing of treatment. Knee Surg Sports Traumatol Arthrosc. 1995;3:130–4. 10.1007/BF01565470 8821266

[22] Anstey DE, Heyworth BE, Price MD, et al. Effect of timing of ACL reconstruction in surgery and development of meniscal and chondral lesions. Phys Sportsmed. 2012;40:36–40. 22508249 10.3810/psm.2012.02.1949

[23] Winkler PW, Csapo R, Wierer G, et al. Sonographic evaluation of lateral meniscal extrusion: implementation and validation. Arch Orthop Trauma Surg. 2021;141:271–81. 33215303 10.1007/s00402-020-03683-1 PMC7886729

[24] Ishii Y, Ishikawa M, Nakashima Y, et al. Visualization of lateral meniscus extrusion during gait using dynamic ultrasonographic evaluation. J Med Ultrason. 2001;2023(50):531–9. 10.1007/s10396-023-01330-x 37286813

[25] Ishii Y, Ishikawa M, Nakashima Y, et al. Knee adduction moment is correlated with the increase in medial meniscus extrusion by dynamic ultrasound in knee osteoarthritis. Knee. 2022;38:82–90. 35930897 10.1016/j.knee.2022.07.011

[26] Shimozaki K, Nakase J, Oshima T, et al. Investigation of extrusion of the medial meniscus under full weight-loading conditions using upright weight-loading magnetic resonance imaging and ultrasonography. J Orthop Sci. 2020;25:652–7. 31590943 10.1016/j.jos.2019.09.009

[27] Guess TM, Razu S, Jahandar H, et al. Predicted loading on the menisci during gait: The effect of horn laxity. J Biomech. 2015;48:1490–8. 25814179 10.1016/j.jbiomech.2015.01.047 PMC4442067

[28] Morrison JB. The mechanics of the knee joint in relation to normal walking. J Biomech. 1970;3:51–61. 5521530 10.1016/0021-9290(70)90050-3

[29] Scanlan SF, Donahue JP, Andriacchi TP. The in vivo relationship between anterior neutral tibial position and loss of knee extension after transtibial ACL reconstruction. Knee. 2014;21:74–9. 23830645 10.1016/j.knee.2013.06.003

[30] Levy IM, Torzilli PA, Warren RF. The effect of medial meniscectomy on anterior-posterior motion of the knee. J Bone Joint Surg Am. 1982;64:883–8. 6896333

[31] Sullivan D, Levy IM, Sheskier S, et al. Medial restraints to anterior-posterior motion of the knee. J Bone Joint Surg Am. 1984;66:930–6. 6736094 10.2106/00004623-198466060-00015

[32] Allen CR, Wong EK, Livesay GA, et al. Importance of the medial meniscus in the anterior cruciate ligament-deficient knee. J Orthop Res. 2000;18:109–15. 10716286 10.1002/jor.1100180116

[33] Garcia SA, Johnson AK, Brown SR, et al. Dynamic knee stiffness during walking is increased in individuals with anterior cruciate ligament reconstruction. J Biomech. 2023;146: 111400. 36469997 10.1016/j.jbiomech.2022.111400

[34] Ishii Y, Okamoto S, Okinaka R, et al. Temporary meniscus extrusion is caused by cumulative stress from uphill and downhill tasks in healthy volunteers. Front Sports Act Living. 2024;6:1271987. 38650839 10.3389/fspor.2024.1271987 PMC11033369

[35] Wang B, Zhong J-L, Xu X-H, et al. Incidence and risk factors of joint stiffness after Anterior Cruciate Ligament reconstruction. J Orthop Surg. 2020;15:175. 10.1186/s13018-020-01694-7 PMC7227360 32410648

[36] Driban JB, Eaton CB, Lo GH, et al. Knee Injuries Are Associated with Accelerated Knee Osteoarthritis Progression: Data from the Osteoarthritis Initiative. Arthritis Care Res. 2014;66:1673–9. 10.1002/acr.22359 PMC4211979 24782446

[37] Ardern CL, Webster KE, Taylor NF, et al. Return to sport following anterior cruciate ligament reconstruction surgery: a systematic review and meta-analysis of the state of play. Br J Sports Med. 2011;45:596–606. 21398310 10.1136/bjsm.2010.076364

[38] Nakamae A, Ochi M, Deie M, et al. Clinical outcomes of second-look arthroscopic evaluation after anterior cruciate ligament augmentation: comparison with single- and double-bundle reconstruction. Bone Jt J. 2014;96-B:1325–32. 10.1302/0301-620X.96B10.34282 25274916

[39] Oiestad BE, Holm I, Aune AK, et al. Knee function and prevalence of knee osteoarthritis after anterior cruciate ligament reconstruction: a prospective study with 10 to 15 years of follow-up. Am J Sports Med. 2010;38:2201–10. 20713644 10.1177/0363546510373876

[40] Driban JB, Ward RJ, Eaton CB, et al. Meniscal extrusion or subchondral damage characterize incident accelerated osteoarthritis: Data from the Osteoarthritis Initiative. Clin Anat N Y N. 2015;28:792–9. 10.1002/ca.22590 PMC4679290 26149125

[41] Murakami T, Enokida M, Kawaguchi K, et al. Useful ultrasonographic evaluation of the medial meniscus as a feature predicting the onset of radiographic knee osteoarthritis. J Orthop Sci. 2017;22:318–24. 28034603 10.1016/j.jos.2016.11.021

